# Fall Detection: Using Technology to Prevent Falls and Save Lives

**DOI:** 10.1093/geroni/igab046.2586

**Published:** 2021-12-17

**Authors:** Lydia Manning

**Affiliations:** Dele Health Tech, United States

## Abstract

Companies, providers, and consumers alike are increasing the use of technology in almost every industry. This increase in usage occurs simultaneously in a technology marketplace characterized by rapid evolution in design and products. Recent years have witnessed the surge of various technological products and solutions in the age-tech marketplace, particularly in senior living and many related to fall detection and prevention interventions. Falls can have a widespread and significant impact on health, can be deadly, and often result in high costs for individuals and older adult living facilities. One out of four older adults fall each year. Findings from a pilot project illustrate the total incident count for a 12-month period. Findings demonstrate the importance of having a complete solution for falls and a fall detection solution in place in an assisted living environment. Ideal environments for residents, families, staff, and those working in the facility with regard to smart tech are considered. It is important to consider how can these solutions empower residents and afford people autonomy and safety through dignified technology.

